# P-722. Impact of Doxy-PEP on Chlamydia and Gonorrhea Percent Positivity at a Large Sexual Health Clinic in the Bronx, New York

**DOI:** 10.1093/ofid/ofaf695.933

**Published:** 2026-01-11

**Authors:** Grace Schuler, Caroline E Mullis, Eric Meyerowitz

**Affiliations:** Albert Einstein College of Medicine, Bronx, NY; Albert Einstein College of Medicine, Bronx, NY; Montefiore Medical Center / Albert Einstein College of Medicine, Bronx, New York

## Abstract

**Background:**

The 2024 CDC Guidelines recommend doxycycline post-exposure prophylaxis (doxy-PEP) to prevent chlamydia (CT), syphilis and gonorrhea (NG) infections gay, bisexual, and other men who have sex with men (MSM) and transgender women (TGW) with a history of a bacterial STI in the past 12 months. Real-world data for doxy-PEP are needed.

**Figure:**
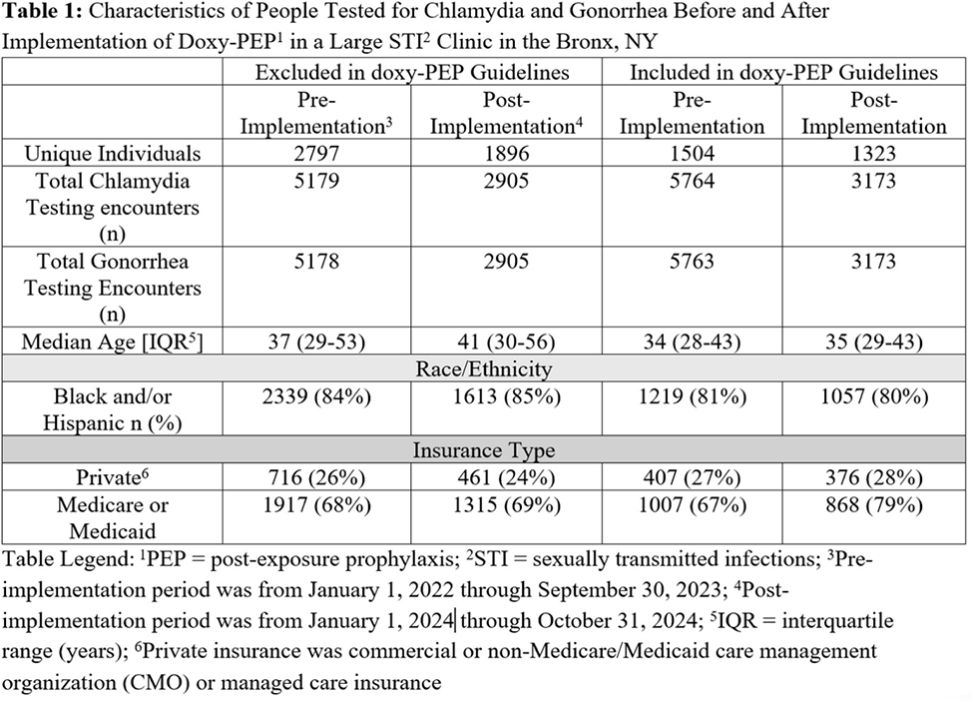

**Figure:**
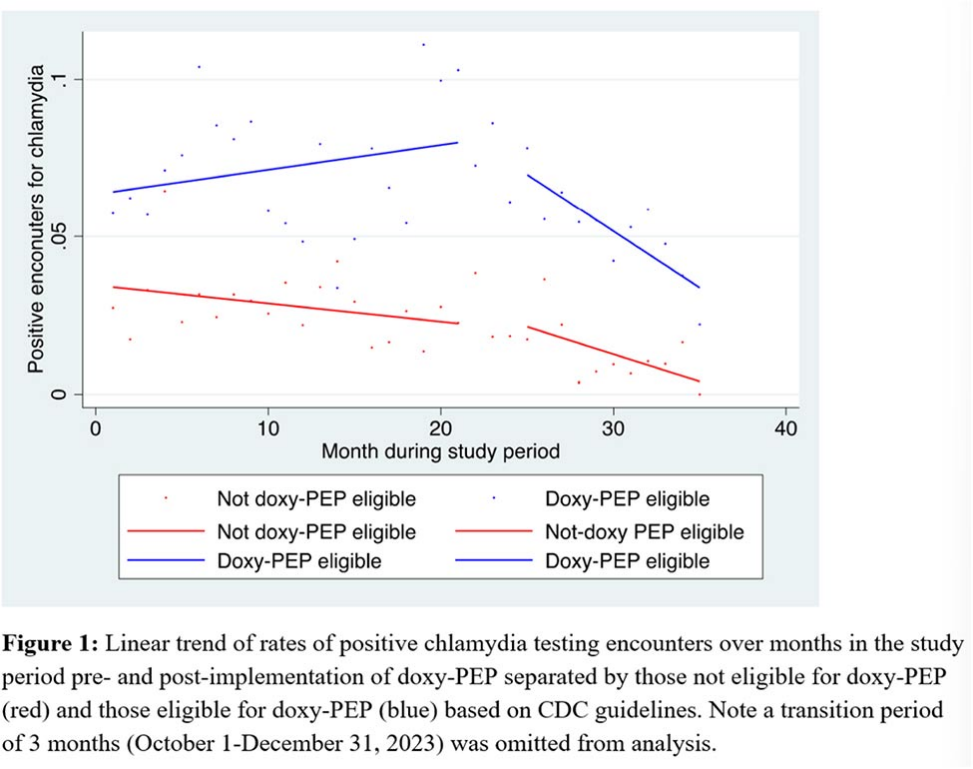

**Methods:**

In this retrospective cohort study, we extracted clinical data for all patients with CT and NG testing at The Oval Center at Montefiore (TOCM), a large sexual health clinic in the Bronx, NY. Patients were grouped as either included in or excluded from the doxy-PEP guidelines based on a sex and gender assignments, MSM designation, and history of rectal swab test in the electronic medical record. Pre- and post-doxy-PEP implementation periods were designated based on timing of TOCM staff training. Pre-post percent positivity was compared using chi-squared tests, and rate of change of percent positivity was calculated using interrupted time series analysis.

**Figure:**
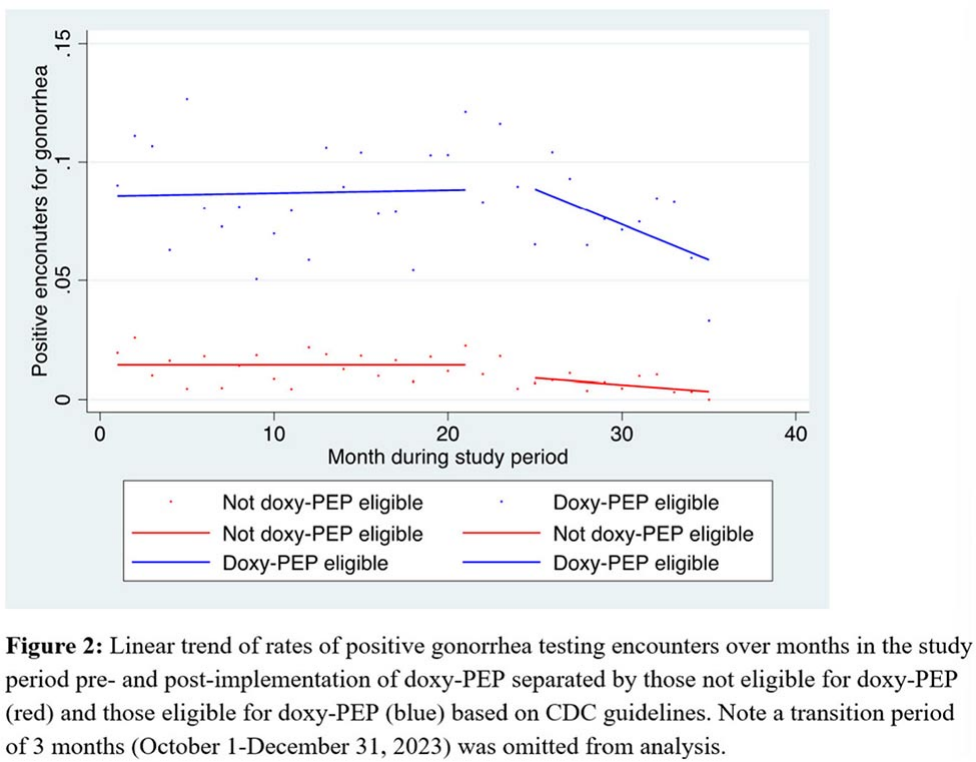

**Results:**

The cohort included 4,322 and 3,180 unique patients in the pre- and post- periods (Table 1). CT positivity declined from 5.1% (563/10,943) to 3.4% (208/6,078) (p< 0.001), 7.2% (416/5,764) to 5.4% (170/3,169) (p< 0.01), and 2.8% (147/5,179) to 1.3% (38/2,909) (p< 0.001), and NG positivity declined from 5.3% (577/10,941) to 4.3% (261/6078, p < 0.01), 8.7% (502/5763) to 7.6% (242/3173, p=0.08), and 1.4% (75/5178) to 0.6% (19/2905, p < 0.01) in the pre- and post- periods for the total cohort, those included, and those excluded from the CDC guidelines.

In the post-period, CT positivity decreased by -0.17% per month (95% CI, -0.348%, 0.002%, p 0.05) and by -0.36 % per month (95% CI -0.54%, -0.18%, p < 0.001) in participants not included and included in the doxy-PEP guidelines, respectively. In the post-period, NG positivity decreased by -0.05% per month (95% CI -0.11%, -0.004, p =0.03) in those not included in doxy-PEP guidelines and was not statistically significant in those eligible for doxy-PEP (-0.30%, 95% CI -0.70, 0.10, p=0.14).

**Conclusion:**

In this real-world analysis of individuals at a sexual health clinic in the Bronx, we found CT positivity declined after doxy-PEP implementation in all groups, and more for those included in the CDC guidelines.

**Disclosures:**

Eric Meyerowitz, MD, Mannkind Corporation: Grant/Research Support|Paratek: Grant/Research Support|UpToDate: Honoraria

